# Exploring the feasibility, effectiveness, and acceptability of telehealth for delivering a pain management group program: A retrospective study

**DOI:** 10.1371/journal.pone.0325298

**Published:** 2025-05-30

**Authors:** Ramony Chan, Bernadette Brady, Judy Zou, Matthew McMullan, Sandhiya Ali, Subram Naidu, Renata Bazina

**Affiliations:** 1 University of New South Wales, Sydney, New South Wales, Australia; 2 Department of Pain Medicine, Liverpool Hospital, Sydney, New South Wales, Australia; 3 University of Sydney, Sydney, New South Wales, Australia; 4 Western Sydney University, Sydney, New South Wales, Australia; University of Minnesota School of Dentistry, UNITED STATES OF AMERICA

## Abstract

**Objective:**

The COVID-19 pandemic caused significant changes in healthcare, particularly in pain management. To maintain care, the Multidisciplinary Activity Improvement Program (MAiP), at the Department of Pain Medicine, Liverpool Hospital, was adapted for delivery through telehealth. Although MAiP’s effectiveness is well-documented, its telehealth adaptation has not been studied. This study retrospectively assesses the feasibility, effectiveness trends, and acceptability of a telehealth-based pain program.

**Methods:**

Using a single-group retrospective cohort design, participants were patients who completed the telehealth-based MAiP between 2020 and 2022. Primary outcomes: pain severity, pain interference, anxiety, stress, depression, pain self-efficacy, and pain catastrophising, were evaluated pre- and post-treatment. Participants’ satisfaction with the program was assessed through a post-program survey. Data analysis employed a generalised estimation equation modelling technique.

**Results:**

33 patients were enrolled in a telehealth MAIP during the study period, with outcomes available for 22 patients (68% female, mean age 51.45 ± 10.41, 72.7% with pain duration >5 years). Significant improvements were observed in the primary outcome measures, indicating the effectiveness of the telehealth-based MAiP. Standardised effect sizes (ES) for all outcomes ranged from small to large. Of the 22 participants, 14 completed the satisfaction survey, with the majority expressing satisfaction and finding the telehealth-based program beneficial.

**Conclusion:**

The study’s findings present initial evidence for the effectiveness and acceptability of delivering pain management group programs via telehealth, expanding the range of services available to patients. These promising results advocate for continued exploration of telehealth as a vital avenue for pain management service delivery, warranting further investigation and advancement in this evolving field.

## Introduction

Since its emergence in December 2019, the global impact of the coronavirus disease (COVID-19), has been profound [1]. This impact extends far beyond the loss of lives and includes economic repercussions, increased poverty rates [1], deteriorating mental health [2], altered lifestyle behaviours [3], challenges in education, and transformations in society at large [4,5]. One of the most significant consequences has been the necessity to adapt healthcare delivery systems to accommodate COVID-19 restrictions, leading to a surge in telehealth adoption [6]. Telehealth, defined as the remote provision of healthcare services without direct physical contact with patients [6,7], encompasses various modalities, including real-time video consultations, asynchronous video clips, mobile health through wearable devices, and electronic health utilising electronic-recorded education [8].

As COVID-related lockdown restrictions limited in-person healthcare delivery, pain clinics faced unprecedented challenges. The adoption of telehealth in pain medicine became not only necessary but also a potential permanent mode of healthcare delivery [8]. Prior to the emergence of COVID-19, internet-based self-guided or clinician-guided individual-based pain management programs had demonstrated their efficacy and garnered patient satisfaction [9–14]. These programs offer valuable alternatives to, though not replacements for, in-person pain management programs, as some patients prefer and benefit more from face-to-face interactions. To continue providing face-to-face group-based pain management programs during COVID restrictions, synchronous telehealth, specifically videoconferencing, can facilitate face-to-face contact between participants and clinicians without physical presence requirements.

The Department of Pain Medicine at Liverpool Hospital, South Western Sydney Local Health District (SWSLHD), Sydney, Australia, adopts a step-care approach to provide tailored pain management services [15–17], incorporating three levels of treatment: the Pain Introduction Day (PID), One Day Education Program (ODEP), and Multidisciplinary Activity Improvement Program (MAiP). It is recognised that while some patients may only require educational support for pain management, others would need more intensive treatment [15,16]. The PID is a two-hour program led by a pain specialist and nursing staff, offering general information about departmental services and education on the biology and medical management of chronic pain. In contrast, the ODEP is a three-hour program facilitated by a pain physiotherapist, a pain clinical psychologist, and nursing staff, focusing on the physiotherapy and psychological aspects of chronic pain. Patients who complete the PID and ODEP and seek further treatment can participate in the MAiP.

The MAiP is a multidisciplinary pain management program that integrates Acceptance and Commitment Therapy (ACT) and Cognitive Behavioural Therapy (CBT). This clinician-guided manualised program is delivered in a group format (eight to ten participants) over eight weeks. A pain clinical psychologist and a pain physiotherapist each lead three-hour weekly sessions, totalling six hours per week and forty-eight hours over the program’s duration. Clinicians utilise a treatment manual to guide the teaching and practice of pain management strategies, employing direct demonstration, group practice, and group discussion. Participants are provided with a program booklet and are expected to actively participate in the program by practicing these strategies at home and providing feedback during each weekly session. In contrast to the educational focus of the PID and ODEP programs, the MAiP prioritises skill development.

During the COVID-19 pandemic, the Department of Pain Medicine has embraced the synchronous telehealth mode for delivering the PID, ODEP, and MAiP. This approach utilised the Pexip software, a video conferencing platform that allows participants to connect in a virtual meeting room integrated into the hospital’s computer system. The format of the PID and ODEP was not changed. However, the MAiP was adapted to an online format, referred to as “online-MAiP,” running for 5 hours per week over eight weeks for a total of 40 hours, with a reduced capacity of 5 patients. Participants received program materials by mail before starting the online MAiP, logging in from home while clinicians accessed the platform from their offices to conduct the sessions. As part of the Electronic Persistent Pain Outcomes Collaboration (ePPOC) data collection [18], all participants were required to complete pre- and post-program questionnaires. These assessments included healthcare utilisation, medication use, the Brief Pain Inventory-Short Form (BPI) [19], the Depression Anxiety Stress Scale-21 (DASS21) [20], the Pain Self-Efficacy Questionnaire [21], and the Pain Catastrophising Scale [22]. The results of these questionnaires were stored in the epiCentre database at the Australian Health Services Research Institute, University of Wollongong [18].

The effectiveness of face-to-face MAiP has been established in previous research [23,24]. It was found that pre-post treatment effect sizes (Cohen’s *d*) across a range of psychological and pain-related outcomes ranged from small to medium [24]. Specifically, the largest improvements were observed in pain distress, maladaptive pain cognitions (such as beliefs about pain causing disability and the possibility of a “cure”), pain self-efficacy, anxiety, and stress [24]. However, the critical question of whether transitioning MAiP to a telehealth format maintains its effectiveness has yet to be addressed. Although existing literature suggests comparable outcomes for telehealth versus in-person group interventions for psychological difficulties [25], it is important to evaluate this specifically within the context of MAiP. This study aims to investigate the effectiveness, acceptability, and participant satisfaction of the online-MAiP. Should the online-MAiP achieve similar effect sizes as its face-to-face counterpart, it could offer an additional treatment modality for patients with chronic pain who face physical attendance challenges at pain centres.

## Materials and methods

### Design

A single-group retrospective cohort design was utilised. The outcome assessments were conducted before and after participation in the online MAiP. Participant outcomes were assessed using the ePPOC questionnaire as part of routine care, and data were stored in the epiCentre, a software program, at Liverpool Hospital. The Human Research Ethics Committee (HREC), SWSLHD, Sydney, Australia, approved this study under the registration number [2022/ETH01047]. The study was conducted according to the ethical standards of Liverpool Hospital and University of New South Wales, Sydney, NSW, Australia.

The recruitment period for this study began on 1 March 2023 and concluded on 31 August 2023. Before enrolment, all participants received an invitation letter, a participant information sheet, and a consent form. Only individuals who read the information sheet were eligible to participate. Informed consent was considered valid once participants returned a signed consent form. By providing written consent, participants agreed both to participate in the study and to grant the researchers access to their ePPOC outcome data stored in the epiCentre. Only the chief investigator, who is also the first author of this study, accessed the epiCentre to retrieve non-identifiable data for research purposes on 4 September 2023. All identifiable and non-identifiable data were stored separately, with each participant assigned a unique code to link the two types of data. Throughout the study, only the chief investigator had access to the identifiable data.

### Participants

A file review was performed for all participants who completed the online MAiP program between 2020 and 2022 at the Department of Pain Medicine, Liverpool Hospital. Eligibility for the online program was determined by the multidisciplinary team (MDT) after a modified MDT assessment (imposed by the pandemic) and included adults (≥18 years) with persistent pain symptoms (≥ 6 months) who had been screened as ineligible for surgical or other interventional procedures for pain management within the next 6-months. All patients had to report a willingness to participate in an 8-week program at the specified dose, have availability of in-home or mobile internet, availability of a device for video conferencing (mobile phone, tablet, computer or laptop) and be able to read, write and speak English. Patients were ineligible for the online MAiP if they were assessed as palliative, diagnosed with cancer-related pain, were unable to speak or understand English, or were unable to connect to the telehealth platform during one of the assessment sessions.

### Measures

This study evaluated several primary and secondary outcomes. Primary outcomes encompassed pain intensity, pain interference, depression, anxiety, stress, pain catastrophising, and pain self-efficacy. Secondary outcomes focused on medication use, specifically the number of major drug groups, daily morphine equivalent dosage (mg), and the frequency of opioid medication use (more than two days per week). This study did not assess participants’ use of non-prescribed opioids. All outcomes were assessed using the electronic Persistent Pain Outcomes Collaboration (ePPOC) questionnaire [18]. This questionnaire incorporated established and validated scales for the primary outcomes: the Brief Pain Inventory-Short Form (BPI-SF), the Depression Anxiety Stress Scale-21 (DASS21), the Pain Self-Efficacy Questionnaire (PSEQ), and the Pain Catastrophising Scale (PCS). The selection of these scales was the result of a rigorous process involving a panel of chronic pain researchers and clinicians from Australia and New Zealand. Through a series of workshops and test trials, the panel prioritised scales demonstrating clinical utility, robust validity and reliability, sensitivity to change in chronic pain populations, good acceptability and low burden for both patients and staff, low or no cost, and availability in multiple languages [18]. Further details regarding the development of the ePPOC questionnaire are available elsewhere [18,26].

#### Brief Pain Inventory-Short Form (BPI-SF).

The BPI-SF is a self-reported measure that assesses four items of pain severity (pain severity sub-scale) and seven items of pain interference (pain interference sub-scale). Each item is rated on a numeric scale from 0 to 10, where higher scores indicate more severe pain or more significant interference [19]. The scale has high internal consistency and a consistent two-factor structure [27].

#### Depression Anxiety Stress Scale – 21 (DASS21).

The DASS-21 consists of 21 questions grouped into three subscales: depression, anxiety, and stress (7 items each). Each question is rated on a scale of 0–3, with higher scores indicating greater severity. The scores for each subscale are summed and multiplied by 2 to calculate the total score. The DASS-21 has been shown to have strong psychometric properties in both general and clinical populations [20].

#### Pain Self-Efficacy Questionnaire (PSEQ).

The PSEQ is a 10-item scale developed to assess the confidence of individuals with chronic persistent pain in carrying out various activities or life roles despite their pain. Each item is rated on a scale from 0 (“not at all confident”) to 6 (“completely confident”), with higher scores indicating greater self-efficacy. The scale has shown strong psychometric properties [21].

#### Pain Catastrophising Scale (PCS).

The PCS, a 13-item scale, prompts participants to recall past painful experiences and then evaluate the frequency of 13 specific thoughts and feelings. Using a 5-point Likert scale ranging from 0 (“not at all”) to 4 (“all of the time”), individuals indicate the degree to which they experienced each item. The PCS yields scores for three subscales – Rumination, Magnification, and Helplessness – calculated by summing responses to the items within each subscale. A total catastrophizing score is derived from the sum of all 13 items. Elevated scores on the PCS indicate a greater degree of catastrophising. Psychometric properties and scale development details are available in the work of Sullivan and colleagues [22].

#### Program satisfaction.

Participants’ program satisfaction and acceptability were evaluated through four different questions previously used in other studies [e.g., 28]. The questions were “Overall, how satisfied were you with the online MAiP program?”; “How has participating in the online MAiP program affected your confidence in managing chronic pain?”; “Would you confidently recommend the online MAiP program to a friend?”; and “Was participating in the online MAiP program worth your time?”. Participants’ responses to the first two questions were rated on a 5-point Likert scale. The last two questions required a simple ‘Yes’ or ‘No’ answer.

### Statistics

SPSS v27 was employed to run statistical analyses. The main statistic to examine changes before and after treatment was Generalised Estimation Equation (GEE) modelling. GEE accounts for within-subject variability while focusing on the average group effect, using a specified working correlation structure. Unlike traditional mixed linear models, GEE directly models average group-related changes over time [29–31]. GEE analyses included maximum likelihood estimation, an exchangeable working correlation structure, and robust error estimation. Linear models were used for primary outcomes and daily morphine equivalent dosage (mg), while ordinal logistic and binary logistic models were used for the number of major drug groups and opioid medications used more than two days per week, respectively. GEE analyses were performed using an intention-to-treat approach, assuming that the data were missing at random. Pre-treatment values were used as baseline measurements for both primary and secondary outcomes. The Holm-Bonferroni adjustment was used to control the familywise error rate at 0.05 [32]. For the primary outcomes, effect sizes were calculated. The advantages of reporting effect sizes, the size of differences between outcomes, are that they better inform the direction and magnitude of treatment effects in addition to the *P* value analysis and facilitate comparison between comparable studies [33]. With a power of 0.80, an alpha level of 0.05, and a sample size of n = 20, the study had adequate power to detect clinical changes associated with Cohen’s *d* effect sizes [34] greater than 0.55.

## Results

### Participant characteristics

A total of 33 patients completed the online MAiP program between 2020 and 2022 and were thus eligible for inclusion in this study. However, complete outcome data were available for only 22 participants; the remaining 11 were excluded due to incomplete questionnaires (n = 10) or excessive missing data (n = 1). Furthermore, only 14 of the 22 participants with complete outcome data returned the post-program satisfaction survey. Reasons for non-completion included time constraints (n = 4), loss of the completed survey via mail (n = 2), and declining to participate (n = 2). This sample size of 22 is similar to the sample size reported in the previous study of the face-to-face MAiP program [24]. Participants maintained an average attendance rate of 94% (ranging from 63% to 100%).

Table 1 presents the participants’ characteristics. Most participants (68.2%) were female, with a mean age of 51.45 (±10.41). Many participants were non-insurance cases (77.3%) and unemployed (81.9) due to health or chronic pain conditions. The average number of pain sites was 16.2 (± 7.67). Additionally, 72.7% of participants had experienced pain for more than five years, and 81.8% reported both physical and mental health problems. More than half of the participants (59.2%) used three or more drug groups, and 54.5% used opioid medications more than two days a week. The mean daily oral morphine equivalent dosage was 23.82 (±30.91) mg.

**Table 1:** Participants’ Characteristics.

	(n-22)	
	n	%
**Gender**		
** Female**	15	68.2%
** Male**	7	31.8%
**Age**		
** Mean (SD)**	51.45 (±10.41)	
** Range**	26–67	
**Insurance status**		
** Yes**	5	22.7%
** No**	17	77.3%
**Number of Pain Sites**		
** Mean (SD)**	16.2 (7.67)	
** Range**	3–32	
**Duration of Pain**		
** Less than 3 months**	0	0%
** 3–12 months**	1	4.5%
** 12 months to 2 years**	1	4.5%
** 2–5 years**	4	18.2%
** More than 5 years**	16	72.7%
**Pain Chronicity**		
** Always present (always the same intensity)**	3	13.6%
** Always present (Level of pain varies)**	18	81.8%
** Often present (pain free periods last less than 6 hours)**	1	4.5%
** Occasionally present**	0	0%
** Rarely present**	0	0%
** Pain is no longer present**	0	0%
**Health Condition**		
** Neither**	0	0%
** Mental health problems only**	2	9.1%
** Physical health problems only**	2	9.1%
** Both mental and physical health problems**	18	81.8%
**Employment Status**		
** Working full time**	1	4.5%
** Working part time**	1	4.5%
** Unable to work due to a condition other than pain**	7	31.9%
** Unable to work due to pain**	11	50%
** Not working by choice**	1	4.5%
** Seeking employment**	1	4.5%
**Medication: Major Drug Group (0–7)**		
** 0**	1	4.5%
** 1**	2	9.1%
** 2**	6	27.3%
** 3**	5	22.7%
** 4**	5	22.7%
** 5**	3	13.8%
** 6**	0	0%
** 7**	0	0%
**Daily Morphine Equivalent Dosage (mg)**		
** Mean (SD)**	23.82 (±30.91)	
** Range**	0–100	
**Opioid Medications >2 days/ wk**		
** Yes**	12	54.5%
** No**	10	45.5%

### Treatment satisfaction

Fourteen of the twenty-two participants (63.64%) completed the program satisfaction survey. 85.71% of participants indicated they were either very satisfied or satisfied with the online MAiP program. No participant felt dissatisfied. Eighty-six per cent of participants reported that the program greatly increased or increased their confidence in managing chronic pain, with no participants reporting a decrease in confidence. Furthermore, 86% of participants would recommend the treatment to others. All participants found the online MAiP program worth their time.

### Primary outcomes

Table 2 shows the primary outcomes’ pre-treatment and post-treatment means and standard deviations. Table 3 presents standard effect sizes (ES), confidence intervals, p-values, and Holm-Bonferroni Adjusted p-values for primary outcomes. The GEE analyses revealed significant effects of time on pain intensity (Wald’s ꭓ^2^ = 21.09, *p*=<0.001), pain interference (Wald’s ꭓ^2^ = 21.26, *p*=<0.001), depression (Wald’s ꭓ^2^ = 16.40, *p*=<0.001), anxiety (Wald’s ꭓ^2^ = 14.82, *p*=<0.001), stress (Wald’s ꭓ^2^ = 10.19, *p* = 0.001), pain catastrophising (Wald’s ꭓ^2^ = 30.57, *p*=<0.001), and pain self-efficacy (Wald’s ꭓ^2^ = 18.63, *p*=<0.001). Effect sizes ranged from small to large, based on Cohen’s *d* effect sizes guideline [34]. Specifically, small effect sizes were found for anxiety (ES: 0.44, 95% CI: 0.26–0.62), medium effect sizes for depression (ES: 0.64, 95% CI: 0.37–0.91), stress (ES: 0.66, 95% CI: 0.31–1.01), and pain intensity (ES: 0.76, 95% CI: 0.46–1.06). Large effect sizes were observed for pain catastrophising (ES: 0.92, 95% CI: 0.68–1.16), pain self-efficacy (ES: 1.16, 95% CI: 0.65–1.66), and pain interference (ES: 1.01, 95% CI: 0.63–1.39).

**Table 2:** Means and standard deviations for the observed means of primary outcomes.

	Estimated marginal means	
	Pre-treatment	Post-treatment
**Depression**	24.7 (12.49)	16.30 (11.74)
**Anxiety**	17.10 (8.52)	12.80 (8.91)
**Stress**	24.10 (8.32)	17.90 (10.39)
**PCS** ^**†**^	29.80 (11.35)	18.80 (12.41)
** Rumination** ^*^	10.15 (3.42)	6.05 (4.21)
** Magnification** ^*^	5.65 (3.60)	3.75 (3.29)
** Helpless** ^*^	14.00 (5.35)	9.00 (5.89)
**PSEQ** ^**‡**^	19.20 (7.64)	28.10 (7.99)
**Pain Interference**	7.25 (1.38)	5.71 (1.75)
**Pain Severity**	6.71 (1.20)	5.91 (1.50)

Note: Standard deviations are shown in parentheses for the means. Depression, anxiety and stress were measured by the Depression Anxiety Stress Scale 21 items, † Pain Catastrophising Scale, ‡ Pain Self-Efficacy Questionnaire, * Subscales of the PCS.

**Table 3:** Primary outcomes GEE results.

	Standardised Effect Sizes (ES)				
		Confidence	Interval		
	ES	Low	High	*P-value*	*Holm-Bonferroni Adjusted P-value*
**Depression**	0.64	0.37	0.91	<0.001	0.011
**Anxiety**	0.44	0.26	0.62	<0.001	0.011
**Stress**	0.66	0.31	1.01	0.001	0.011
**PSEQ** ^**‡**^	1.16	0.68	1.16	<0.001	0.011
**PCS** ^**†**^	0.92	0.65	1.66	<0.001	0.011
** Rumination** ^*^	1.16	0.84	1.47	<0.001	0.011
** Magnification** ^*^	0.55	0.34	0.75	<0.001	0.011
** Helpless** ^*^	0.88	0.61	1.15	<0.001	0.011
**Pain Interference**	1.01	0.63	1.39	<0.001	0.011
**Pain Severity**	0.76	0.46	1.06	<0.001	0.011

Note: Depression, anxiety and stress were measured by the Depression Anxiety Stress Scale 21 items, † Pain Catastrophising Scale, ‡ Pain Self-Efficacy Questionnaire, * Subscales of the PCS.

### Secondary outcomes

The results of the GEE analyses were not statistically significant on all secondary outcomes: the number of major drug groups (Wald’s ꭓ^2^ = 2.23, *p* = 0.135), daily morphine equivalent dosage (mg) (Wald’s ꭓ^2^ = 2.94, *p* = 0.087), or the use of opioid medications more than two days per week (Wald’s ꭓ^2^ = 0.34, *p* = 0.561).

## Discussion

This study aimed to assess the effectiveness, acceptability, and participant satisfaction of the online-MAiP program, which transitioned to synchronous telehealth delivery during the COVID-19 pandemic. The study results indicate that the online-MAiP program, delivered via synchronous telehealth, effectively improved primary outcomes related to chronic pain management over the treatment period. Significant improvements were observed in pain intensity, pain interference, depression, anxiety, stress, pain catastrophising, and pain self-efficacy. Importantly, these improvements were associated with effect sizes ranging from small to large, indicating the clinical significance of the online-MAiP program. Notably, large effect sizes were observed for pain catastrophising, pain self-efficacy, and pain interference, underscoring the program’s potential to address critical aspects of chronic pain management effectively.

### Transition to synchronous telehealth

The current study’s findings suggest that transitioning to synchronous telehealth had minimal impact, as the observed small to large effect sizes in primary outcomes indicate the maintenance of therapeutic benefits. These effects marginally exceed the small to medium effects shown in a previous face-to-face pain management program [24], which reported small effect sizes on depression, anxiety, stress, and pain self-efficacy. In contrast, the current study observed medium effect sizes on depression and stress, as well as a large effect size on pain self-efficacy. These variations may be due to methodological differences between the studies, such as program content, the psychometric measures used, and the statistical analyses applied. Despite these differences, the comparable overall results between the studies could be attributed to the maintenance of therapeutic alliance and group cohesion in the telehealth format, both significant predictors of treatment outcomes [35,36]. Research suggests that therapeutic alliance and group cohesion can be effectively established in online group therapy [35–39]. Intriguingly, some research even indicates that group cohesion can be stronger in online groups than face-to-face groups [36,38]. Indeed, some patients prefer online group treatment [36,38]. These factors are key predictors of cognitive and behavioural change in online group therapy [35]. Moreover, the high attendance rate in this study likely contributed to mitigating the impact of potential challenges and maintaining therapeutic outcomes. High attendance is associated with better outcomes in online programs [35]. Recent studies have found online group program attendance to be comparable to, and sometimes even higher than, face-to-face programs [35,37,38]. Although no significant negative effects were observed, future research should directly compare online and face-to-face group programs within an RCT, incorporating qualitative and quantitative assessments of therapeutic relationships, group dynamics, group cohesion, and therapeutic engagement.

### Effectiveness of synchronous telehealth

The current study’s results are consistent with previous research on face-to-face pain management group-based programs and internet-delivered self-guided individual-based pain management programs [9,11,13,23,24]. All three service modalities have shown effectiveness over time in delivering pain management programs to patients with diverse needs. However, synchronous telehealth, particularly through videoconferencing, offers several advantages [6–8,13]. It preserves the core elements of face-to-face interactions between patients and clinicians, facilitating multidisciplinary care even when physical presence is not possible.

The synchronous telehealth modality provides a solution for healthcare accessibility, particularly among participants residing in remote locales. This approach, facilitated through videoconferencing, has increased access to pain management group treatment, while also fostering meaningful engagement among participants and clinicians. Previous studies corroborate the equivalence of group dynamics between telehealth-based interventions and traditional face-to-face treatments [25,35–38]. More recent studies suggest that group dynamics in online treatment are not as negatively impacted as previously thought, although they may require more time and enhanced clinician communication skills [37,38,40,41]. Researchers have proposed strategies to enhance group formation and dynamics in telehealth settings, including careful participant screening, pre-group preparation, additional group rules, utilising “breakout rooms” in videoconferencing, attending to individual facial expressions, self-disclosure, and incorporating environmental context [40,41]. Telehealth group treatment, facilitated by appropriate technology and clinicians’ therapeutic skills, then enables participants to interact verbally and non-verbally, share information, and receive support from peers and clinicians [25,36,38]. Therefore, the telehealth approach provides an additional service modality to enhance service delivery of pain management programs.

### Combining CBT and ACT approaches

An outstanding feature of the online MAiP is the combination of Cognitive Behavioural Therapy (CBT) and Acceptance and Commitment Therapy (ACT) approaches. While each approach is individually effective in treating chronic pain [14,42–44], their combination is relatively uncommon in pain management programs [45]. This integrated approach has the potential to enhance treatment efficacy, as the two therapies are not mutually exclusive [46,47].

ACT focuses on developing psychological flexibility and promoting committed action by encouraging acceptance of thoughts and feelings, and focusing on value-driven life changes [47]. In this program, ACT techniques—specifically acceptance, cognitive defusion, and values clarification—were integrated with traditional CBT pain management strategies, including relaxation, pain desensitisation, cognitive restructuring, activity pacing, sleep management, goal setting, flare-up management, and communication skills training.

Integrating acceptance and values-based living proved less challenging than incorporating cognitive defusion. Participants learned to accept the presence, chronicity, and nature of their chronic pain through metaphors and mindfulness exercises. Values clarification, within the CBT goal-setting framework, helped participants identify personally meaningful goals aligned with their values, thereby supporting committed action.

The integration of cognitive defusion and restructuring was carefully considered. The literature suggests that cognitive defusion can be a valuable complement to cognitive restructuring, particularly in situations where restructuring is challenging or inappropriate [46,47]. Firstly, some cognitions, especially in chronic pain, are accurate and difficult to directly challenge or restructure. For instance, statements like “my pain is always there” or “I am in pain” reflect reality, making traditional cognitive restructuring less effective and potentially leading to experiential avoidance. Second, some patients may be resistant to cognitive challenging, finding it confronting or perceiving it as an invalidation of their experiences. Cognitive defusion provides an alternative approach by helping patients create distance from these cognitions, allowing them to engage in committed actions without necessarily changing the content of their thoughts [47].

The program initially introduced cognitive restructuring, followed by cognitive defusion. Participants were taught that cognitive challenging techniques are preferred when restructuring is feasible; however, when struggling with restructuring, they could utilise cognitive defusion. Despite the advice, participants were free to choose and utilise any techniques that best suited their needs. Interestingly, participants reported that practising cognitive defusion reduced the perceived believability of their thoughts, making them easier to challenge and change.

This synergistic combination of CBT and ACT, while complex, holds significant promise for improving the effectiveness of pain management programs [45]. Future studies may explore whether the utilisation of both approaches indeed improves the effectiveness of the program.

### Technological challenges

Conducting synchronous telehealth-based pain management group programs comes with its share of technological challenges, for example loss of control over the therapeutic setting, diminished therapist presence, reduced physical presence, and increased distractions and interruptions [40]. Issues with computer availability, stable internet connections, and participants’ familiarity and confidence with using technology are important considerations and have been reported by previous research [7,48–52]. These challenges are more prominent in regions where there is a higher level of social disadvantaged population [48]. It is important to provide participants with ongoing support for utilising telehealth and overcoming technological challenges when delivering a telehealth group-based program. Moreover, the capabilities of the telehealth platform itself play a pivotal role in program success [25,48,49,51]. A simple computer program for streaming video and audio may be sufficient for telehealth individual-based consultation. However, to facilitate education, group interaction and small group discussion in a telehealth group-based program, a computer software would need to provide many functionalities, such as screen sharing, whiteboard, break-out room, instant messaging and recording capacity. Despite these challenges, the online MAiP was successfully delivered on many occasions. Improving these technological challenges would enhance the success of online programs.

### Study limitations and strengths

The limitations of this study include its retrospective design, the absence of a control group, small sample size, and the lack of long-term follow-up data. While comparable to the sample size in the previous face-to-face MAiP study [24], the small sample size limits the statistical power to detect smaller effects and may impact the generalisability of the findings. Larger, more adequately powered studies are needed to confirm these preliminary results and explore potential subgroup effects. Caution is needed when interpreting the results due to these limitations. Furthermore, the non-completion of ePPOC measures represents a typical challenge in online programs, and this was also observed in the current study [e.g., [53],^54^]. The online format inherently complicates the monitoring of assessment completion by clinicians. A study highlighting this difficulty in a telehealth group program during the COVID-19 pandemic documented a non-completion rate of more than thirty-five per cent for a web-based questionnaire [53]. This finding is comparable to the current study results and aligns with the broader literature indicating lower response rates for web-based surveys [53,55]. Therefore, future research should explore and implement strategies to improve the completion of outcome measures in telehealth-based treatment programs. Nevertheless, this study offers essential support for conducting large-scale randomised controlled trials (RCTs) to evaluate the effectiveness of synchronous telehealth-based pain management group programs.

On the other hand, the study’s strengths are notable. It demonstrates the feasibility of synchronous telehealth service delivery as an effective alternative to face-to-face approaches for delivering pain management group programs. The study achieved good clinical outcomes and high patient satisfaction, despite the challenges encountered during the online MAiP. It underscores the importance of treatments being both effective and acceptable to patients. Moreover, the study highlights the potential of utilising both CBT and ACT approaches in pain management programs.

## Conclusion

The present study, while preliminary, suggests that synchronous telehealth-based and combined CBT-ACT pain management programs are both acceptable and effective. The online program demonstrated comparable improvements to the face-to-face version, indicating its potential to expand access to much-needed pain management services. Future research should focus on replicating these findings in larger, more controlled trials and examining the long-term clinical impact of this accessible intervention.

## Supporting information

S1 Online MAiP Minimaldataset Data Management. (DOCX)

S2 Online MAiP Minimal Dataset. (XLSX)
