# Supplementary material for: Exploring the feasibility, effectiveness, and acceptability of telehealth for delivering a pain management group program: A retrospective study
Source: PLoS One. 2025 May 30;20(5):e0325298. doi: 10.1371/journal.pone.0325298 (PMC12124518; doi:10.1371/journal.pone.0325298)
Supplement: S1 Online — (DOCX) [file pone.0325298.s001.docx]

**Oline MAiP Research Minimal Data Set Data Management**

| **Variable Name** | **Variable Description and Value** |
| --- | --- |
| ID | Identification number |
| Gender | Gender  0 Male  1 Female |
| AGE | age |
| INS | Insurance status  0 No  1 Yes |
| DP | Duration of pain  1 less than 3 months  2 3-12 months  3 12 months to 2 years  4 2-5 years  5 more than 5 years |
| PC | Pain chronicity  1 Always present (always the same intensity)  2 Always present (level of pain varies)  3 Often present (pain free periods last less than 6 hours)  4 Occasionally present  5 Rarely present  6 Pain is no longer present |
| HC | Health Condition  0 Neither  1 Mental Health  2 Physical Health  3 Both mental and physical health |
| EMP | Employment status  1 working full time  2 working part time  3 unable to work due to a condition other than pain  4 unable to work due to pain  5 not working by choice  6 seeking employment |
| PSIT | Number of pain sites |
| PRMDG | Pre Medication, major drug groups 0-7 |
| PRDM | Pre daily morphine equivalent dosage in mg |
| PROPI | Pre opioid medications > 2 days/ wk  0 No  1 Yes |
| POMDG | Post Medication, major drug groups |
| PODM | Post daily morphine equivalent dosage in mg |
| POOPI | Post opioid medications > 2 days/ wk  0 No  1 Yes |
| PRPSEV | Pre pain severity |
| PRINTER | Pre pain interference |
| PRDEP | Pre depression total score |
| PRANX | Pre anxiety total score |
| PRSTR | Pre stress total score |
| PRRUM | Pre rumination total score |
| PRMAG | Pre magnification total score |
| PRHELP | Pre helplessness total score |
| PRPCS | Pre pain catastrophizing scale total score |
| PRPSEQ | Pre pain self-efficacy questionnaire total score |
| POPSEV | Post pain severity |
| POINTER | Post pain interference |
| PODEP | Post depression total score |
| POANX | Post anxiety total score |
| POSTR | Post stress total score |
| PORUM | Post rumination total score |
| POMAG | Post magnification total score |
| POHELP | Post helplessness total score |
| POPCS | Post pain catastrophizing scale total score |
| POPSEQ | Post pain self-efficacy questionnaire total score |
| OS | Overall satisfaction of the online MAiP  4 Very satisfied  3 Mostly satisfied  2 Neutral  1 Somewhat dissatisfied  0 Very dissatisfied |
| RECOM | Confidence in recommending the online MAiP to a friend  1 Yes  0 No |
| WORTH | Time worth doing the online MAiP  1 Yes  0 No |
| CONFID | Increase confidence in pain management  4 Greatly increased  3 Increased  2 No change  1 Reduced  0 Greatly reduced |
| ATTEND | Attendance (number of sessions) |
| AR | Attendance Rate (%) |
